# A qualitative study of use of long-lasting insecticidal nets (LLINs) for intended and unintended purposes in Adami Tullu, East Shewa Zone, Ethiopia

**DOI:** 10.1186/s12936-018-2209-5

**Published:** 2018-02-06

**Authors:** Zerihun Doda, Tarekegn Solomon, Eskindir Loha, Taye Gari, Bernt Lindtjørn

**Affiliations:** 1 College of Social Sciences & Humanities, Hawassa University, P.O. Box 005, Hawassa, Ethiopia; 2 School of Public and Environmental Health, Hawassa University, Hawassa, Ethiopia; 3 Centre for International Health, University of Bergen, Bergen, Norway

**Keywords:** LLINs, Malaria, Intended uses, Misuses, Repurposed uses, Collateral benefits of LLINs

## Abstract

**Background:**

Malaria poses a significant public health threat globally, across Africa and in Ethiopia. The use of long-lasting insecticidal nets (LLINs) is currently a proven prevention mechanism. Evidence is building on what happens to LLINs following mass distribution campaigns, with mixed results from different studies, some reporting very low use for intended purposes, others an encouraging level of using for intended purposes. In Ethiopia, between 2005 and 2015, about 64 million LLINs were distributed through periodic mass campaigns with the aims to achieve 100% coverage and 80% utilization. However, studies from rural Ethiopia showed variable LLINs coverage and utilization rate. The MalTrial Project, a collaborative venture between Hawassa University, Ethiopia and NROAID, Norway, has started a trial project in 2014 in Adami Tullu District of central Ethiopia. Quantitative surveys have established evidence on LLINs ownership and utilization, but the behavioural, sociocultural and socioeconomic dynamics of why LLINs’ use for intended purposes is low or why they are employed for other purposes remained elusive. The present qualitative study, building on the quantitative findings and framework, therefore, attempted to fill gaps in these areas using qualitative methods in selected localities of the district.

**Methods:**

The study employed 7 focus groups, 16 individual interviews and observation to undertake data collection in January 2017. The data were analysed using NVivo Version 11 (QSR International) to transcribe, code and identify themes using thematic analysis approach.

**Results:**

The study found out that certain households were more likely to use nets for intended needs in proper ways; a range of factors, notably socio-cultural and poverty, highly influence users’ ideas about the right ways and decisions to use and care for the nets; knowledge gaps and wrong perception exist regarding the purposes and life cycle of the nets; LLINs are employed for repurposed uses once they are considered non-viable, old, or lose their physical integrity; existence of misuse was acknowledged and understood as wrong; and values about gender roles further shape uses, misuses and repurposed use of the nets.

**Conclusions:**

Behavioural, socio-cultural, economic and ecological conditions coupled with deficiencies in perceived bed net design and distribution policies; weak education, communication and social support structures were important in understanding and accounting for why a low level of intended use and a rampant misuse and repurposed use in Adami Tullu community of Ethiopia. A major nexus to address in order to improve intended use of LLINs lies, first and foremost, in economic poverty and socio-cultural factors that underlie much of the misuse and repurposed use of the nets.

## Background

Globally malaria is a major threat to about 3.2 billion persons [1], a large burden of disease in the world [2], posing a significant public health threat across Africa [3] and remaining one of the major public health concerns in Ethiopia [4]. The use of long-lasting insecticidal nets (LLINs) is currently a proven prevention mechanism [5], and the most commonly available intervention to prevent the disease in Africa [6], which is also Ethiopia’s key prevention and control strategy [7], and promoted as an effective method for reducing malaria transmission risk. However, the efficacy of LLINs for malaria control depends on a range of factors. Ernst et al. [8] argue that evidence is building on what happens to LLINs following mass distribution campaigns; however, there are mixed results from different studies, some reporting the use of the nets for intended purposes is generally low while other studies arguing most distributed LLINs were used for intended purposes.

In Ethiopia, between 2005 and 2015, about 64 million LLINs were distributed through mass campaigns to achieve 100% coverage and 80% utilization [9]. However, according to 2015 national malaria indicator survey report, only 64% of households (HHs) own at least one LLIN, and 40% of the population slept under the LLINs the night before the survey. The households with at least one LLIN for every two people were 31.7% in 2015. In Oromia region, where this study was conducted, about 58.5% of HHs owned one LLIN and 41% of the population slept under the LLINs the night before the survey [4]. Moreover, studies from rural Ethiopia showed variable LLIN coverage and utilization rate [10]. A recent study by Hailu et al. [11] showed that the proportion of the household with at least one LLIN was only 12%. A study from the Arba Minch area in the Rift Valley in Ethiopia showed the coverage and utilization of LLINs vary through time. Although high coverage was recorded in the study, the mean net utilization remained 20% and 62% before and after the distribution, respectively [12].

The on-going study in the Zwai area of Adami Tullu District of central Ethiopia evaluated bed net utilization on weekly bases for 2 years. The study is part of a project known as MalTrials. The Project is a joint partnership between Addis Ababa and Hawassa Universities from Ethiopia and University of Bergen from Norway. It is a trial project that started in 2014 to study the added effect of combinating long-lasting insecticidal nets and indoor residual spraying compared to using them separately to reduce malaria incidence in Adami Tullu District of central Ethiopia. The results showed that the functional survivorship of LLINs was 79% at 6 months, 39% at 12 months, 13% at 18 months and only 4% at 24 months of follow up. The median (95% CI) serviceable time of functionally surviving LLINs was only 12 (11.6–12.4) months. Of 1491 LLINs that were lost, 43% were thrown away, 21% given away, 15% were torn, and 9.2% were used for other purposes. Meanwhile, potential vector breeding sites were identified and their distance from each household was calculated using ESRI^®^ArcMap™ 9.3 (CA, USA). The distance of households from vector-breeding sites was associated with functional survival of LLINs. Households in more than one km distance from vector breeding sites had 30% risk of losing their LLINs compared to those within one km distance. The first year mean LLINs utilization was 36.2% and the second year was only 4.6% (Solomon et al., pers. comm.). The preliminary results showed that the malaria incidence was in dramatic decline, with only 37% of baseline malaria incidence [13].

The study has established evidence on LLINs survivorship and utilization, but the behavioural, sociocultural and socioeconomic dynamics of why LLINs were employed for misuses and repurposed uses remained elusive. The present study, undertaken after roughly two and half years since the start of quantitative pilot study and LLINs distribution, therefore, attempted to fill gaps in these areas using qualitative methods in selected localities of the district.

## Methods

### Study setting and population

**Fig. 1:**
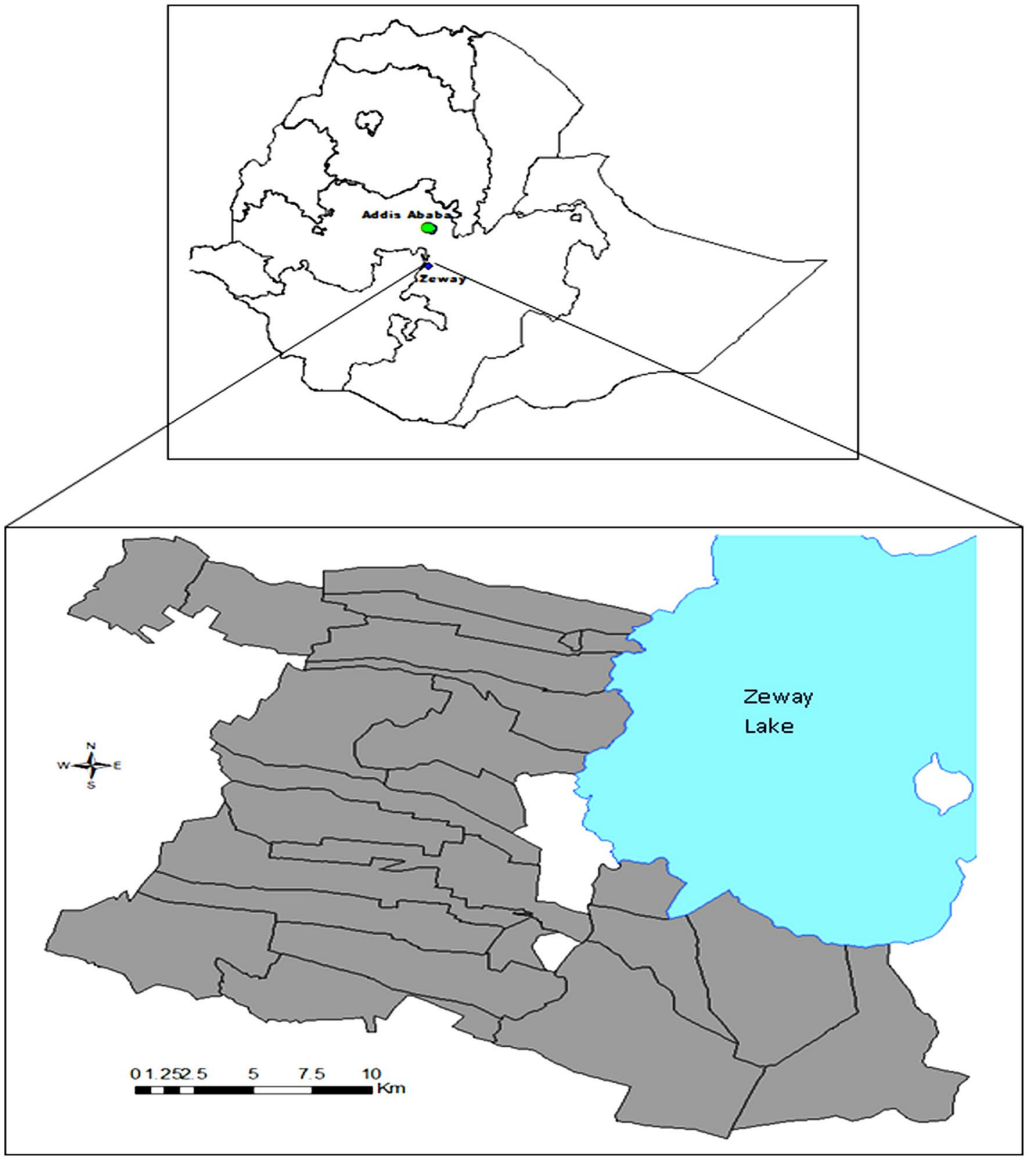
Map of the study area

The study was carried out in Adami Tullu District of Oromia Regional State, south central Ethiopia, located at about 160 km south of Addis Ababa, the national capital (Fig. 1). The fieldwork for the study was undertaken in the first week of January 2017. The District is inhabited by Muslim Oromo. Few other ethnic groups (e.g. Zay) also live intermixed, mostly adhering to Ethiopian Orthodox Christianity. The community practices mixed livelihood activities of crop production, cattle raising and fishing.

Malaria is among the leading causes of health problem in the area, mainly occurring between September and December following the main rainy season in July and August. Moreover, Lake Zeway and irrigation activities around it contribute to mosquito breeding sites and the occurrence of malaria throughout the year.

This qualitative study is a follow up study of the main quantitative-based project (q.v.), in early October 2014, about 7740 LLINs (PermaNet^®^2.0) were distributed for 3006 households in 13 *kebeles* (a term in Amharic, the national language of Ethiopia, that denotes a political-administrative unit) in the district by MalTrials Project. The LLINs were distributed based on family size and the national malaria guidelines recommendation, i.e., ≤ 2 family size = 1 net, 2–5 = 2 nets, 6–7 = 3 nets and ≥ 8, 4 nets. Out of 176 study clusters, 88 were selected for distribution of LLINs. Each cluster had an average of 35 households and was used for unit of randomization for the main study [13]. The households under MalTrials project follow-up did not receive LLINs from 2015 mass campaign.

### Study design

The present study employed cross-sectional, descriptive qualitative methods to understand the contexts and factors of LLINs use, misuse and repurposed use. A total of 7 Focus Group Discussions (FGDs) and 16 individual interviews were conducted. Local users were selected from 5 *kebeles* based on their proximity to mosquito breeding sites and their assignment to malarial treatment plans. Participants in the study were recruited based on a range of criteria, such as permanent residence, having LLINs user experience, being adult household heads, women with breastfeeding babies, local health extension workers, local administration officials and religious leaders.

The breeding site indicator is important in this study. The selection of this variable was based on existence of a major lake and irrigation activities. Breeding sites primarily concern these entities. How closer or farther away households lived vis-à-vis lake shores and irrigation water points was, therefore, important indicator in understanding and evaluating the likelihood of proper use of LLINs. Distance from the breeding sites was thus linked to each of the individual participants according to their residential house location in the selected villages.

### Methods of data collection

#### FGD with local community members

4 women’s and 3 men’s FGDs were conducted. The FGD sessions took maximum of 100 and minimum of 48 min. The size of group participants varied from 8 to 11. Two FGDs were conducted at a locality nearest to vector breeding sites. Overall, 39 women and 30 men participated in the discussion, making a total of 69 participants from the 5 *kebeles*.

#### Individual interviews

11 individual interviews with community members having direct bed net user experience were conducted. Further, 5 key informant interviews with individuals from a range of positions of influence in the community was done. The individual interviews represented informants from a range of backgrounds, along gender, age, religious, proximity to breeding site and user experience.

#### Observation

The verbal data from interviews were further corroborated with visual data. Mainly non-obtrusive and in few cases obtrusive observation were undertaken to document relevant “unsaid” data. These data were linked to such important dimensions as types and extent of misuse and repurposed uses of LLINs, and similar issues. Observation was supported with visual documentation, using a digital sill photo camera.

### Data analysis

The data were recorded using a digital voice recorder. Transcription, management and analysis of the same were done using NVivo Version 11 (QSR International). The data were content-coded for thematic analysis. Initial coding activity was based on prior conceptual categories and further coding concepts were derived from the data. Explorations of coded data were done to make further analytical activities such as querying the data to find out frequently occurring concepts and themes, relationships among codes and themes. The analysis came up with six salient themes (that form the basis for the discussion below).

## Results

### Socio-demographic characteristics

Of the combined total number of informants (N = 86), the majority of user informants were female, adult, Muslim, and living in localities near breeding sites. Of the total study participants, 18 (20.9%) were Christian, the rest (79.1%) were Muslim. Female participants were slightly higher (46, 53.5%) than males, and the majority were within the ages category 25 and 50. Except for the health extension workers, all of the study participants were farmers or fishermen by occupation.

### Use of LLINs for intended purpose: perceptions and experiences

*Informants’ views about benefits* of using LLINs drew two responses: they would almost always first reply that the primary benefit was to protect from malaria and then this would follow with the mention of collateral benefits of the nets, namely, killing and repelling bed bugs and insects. There was almost a sense of fascination with the way the nets would kill other insects and protect sleepers from these nuisances. One informant reported that the primary intended benefit of LLINs was “to get a peaceful sleep”.

**Fig. 2:**
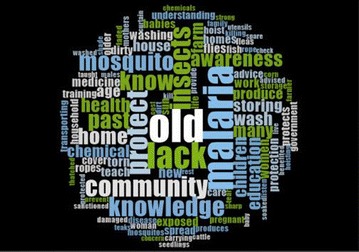
Word Cloud: 50 most occurring words showing terms like “babies”, mothers” and “pregnant” (Produced from NVivo Version 11)

Regarding *knowledge of intended purpose of LLINs*, there was a divided understanding among informants, some reporting that there were certain individuals who lack proper knowledge, while others arguing all people now know about intended purposes. However, the general observation was that knowledge gap exists. Informants who reported either themselves or others they knew who used LLINs for the intended purposes were further asked to identify characteristics of such proper users. The study showed households with babies and pregnant women were category of persons most likely to use LLINs for intended purposes (Fig. 2).

People who lived in a corrugated-iron roof houses were also more likely to use LLINs in proper ways. Interviewees in fact would quite routinely refer to this fact; many reported a key reason for failure to use the nets properly was because their thatched-grass wall and roof house was a key obstacle, as the traditional house with its characteristics windowless and space-limited design often leading to too much soot and dirt. In a sense, thus, type of house is both an indicator of socio-economic status and an important factor influencing use. Proximity to malaria breeding sites and access to mass media were further reported as factors in using LLINs for the intended purposes. Furthermore, some individuals mentioned *expectation of positive sanctions* as a factor as was *fear of economic cost if children get sick following malaria attack*.

### Misuses and repurposed uses

**Fig. 3:**
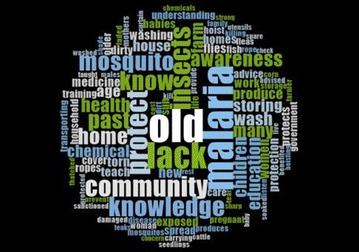
Word Cloud: 100 most occurring words, showing the term “old” as the most frequent one (NVivo Version 11)

Both misuse and repurposed use of LLINs were observed and reported as dimensions of unintended uses. Misuse is when nets are put to other uses during their life span of expected intended purposes. Repurposed use is when people use nets for other purposes after perceived loss of killing power in the nets or when they get old and torn. In general, informants acknowledged that using LLINs for unintended purposes was a fact and pervasive. An interesting question was to find out *when or at what stage of the lifespan* of LLINs did people use the nets for unintended purposes. Most informants reported LLINs were used for other purposes when old, torn up and otherwise lost their physical integrity (Fig. 3). This shows repurposed use generally outweighs misuse of nets.

Certain types of local needs (such as ropes) were generally associated with repurposing, while certain other types of unintended uses (such as clothing, curtains, head scarfs, bed sheets, and blankets) were likely in requiring LLINs in their new forms (see below). Thus, the misuse of LLINs was both acknowledged by the informants as well also generally expected. It was also acknowledged as particularly wrong (compared to repurposed uses).

### Behaviours and practices related repurposed-uses

**Fig. 4:**
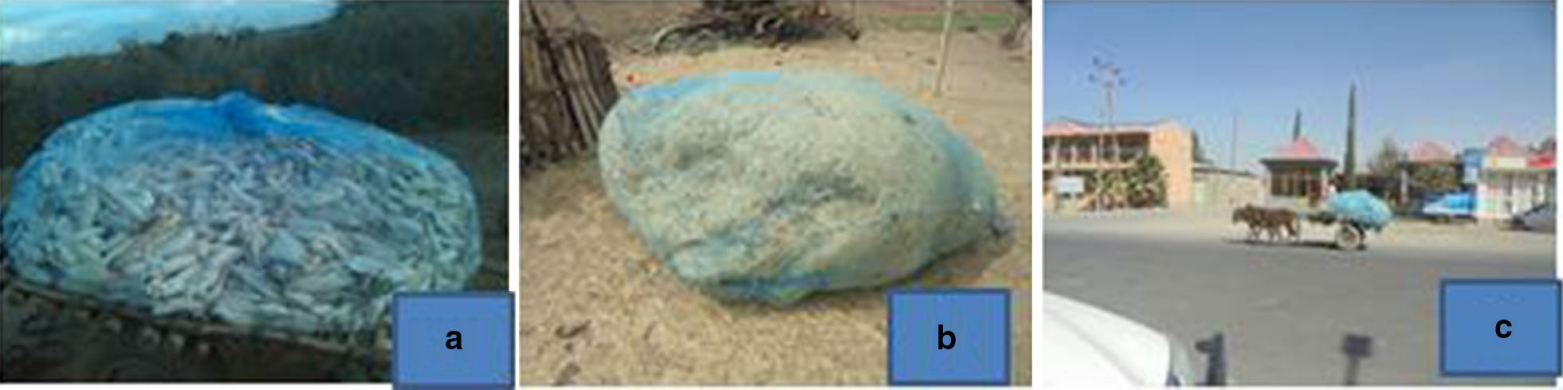
Types of other uses in the “livelihood productive” category. In order appearance: **a** Corn wrapped up; **b** *ţeff* (*Eragrostis tef*) residue wrapped up; **c** people transporting on donkey cart

**Fig. 5:**
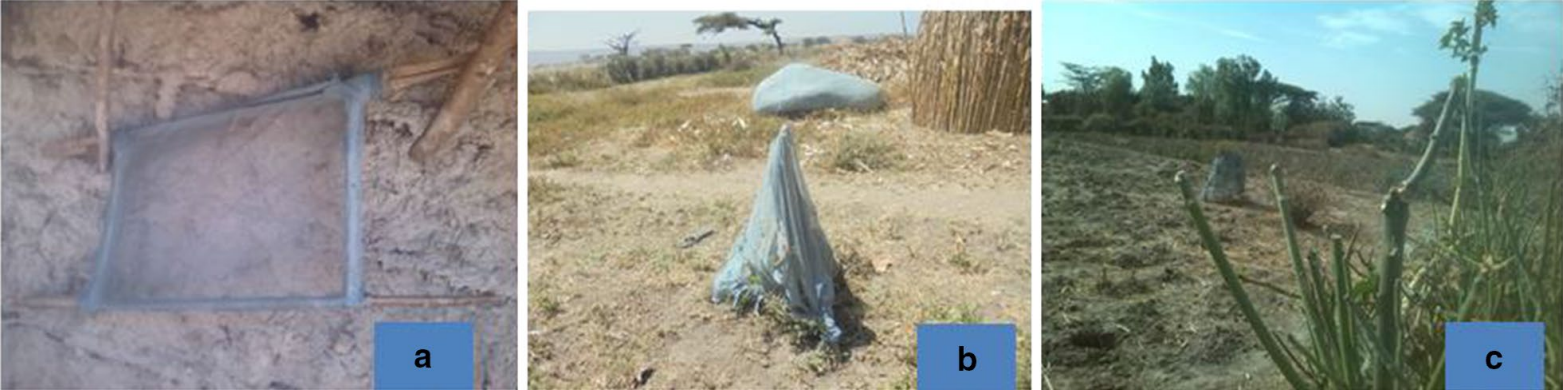
Different other uses: **a** sieving tool; **b** small tree wrapped; corn wrapped, **c** small tree fenced

Productive needs are the most commonly observed repurposed uses, linked with the local communities’ livelihood needs. LLINs thus serve a range of functions, such as storing, transporting and barn topping during harvest seasons (Fig. 4) and sieving sand and *teff* (*Eragrostis tef*) (Fig. 5).

A commonly reported and observed repurposed use was making ropes out of the nets. Ropes are in high demand for variety of needs: tethering livestock, binding firewood, and pulling water from wells. Informants reported locally available row materials used for making ropes were disappearing and that ropes from the LLINs are very robust.

**Fig. 6:**
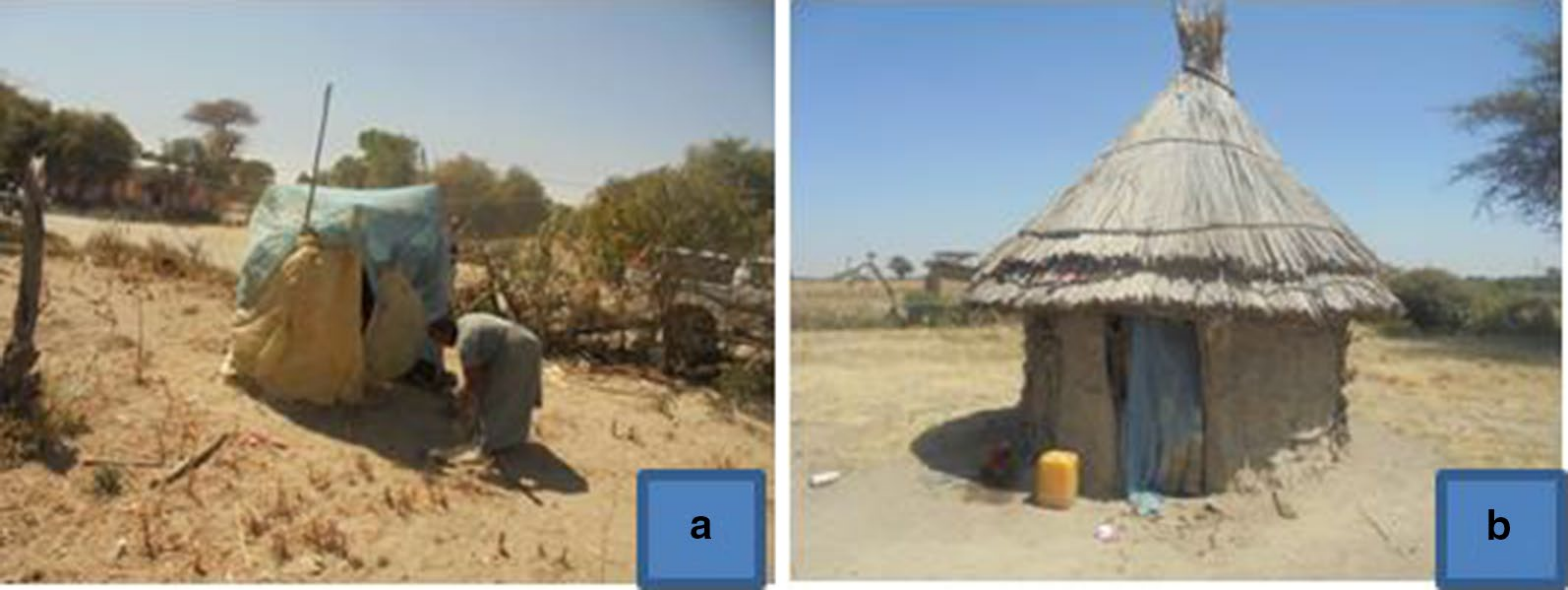
Photos showing LLINs used as toilet roof covers (**a**) and door curtain (**b**)

Another dominant repurposed use related to fishing, which is an important livelihood activity for communities near the Lake. LLINs are used to trap small fish which in turn are used as baits to catch larger fish. LLINs are also used in fencing or as protective material around seedlings and plastering outdoor toilet walls (Fig. 6).

### Behaviours related to misuses

In the household, LLINs are used as bedding supports, such as blankets, bed sheets and mattress covers. Misuse behaviours were more likely in this realm. For example, a common reported misuse behaviour was using the nets as clothing and decorative stuff. Further, the misuse of LLINs for curtains was a common practice. The colour of the net was reported as very appealing for many households. In rare cases, some were reported as misusing the nets for stitching cloths, while others for head scarves. Other reported rarer forms of misuse behaviours include: selling away, giving as gifts to family members living elsewhere and relatives, and simply keeping stored.

### Reasons of misuses and repurposed uses

While reasons may be shared for both misuse and repurposing related behaviours, attention is called to the fact that the two have different motives. Some of the reasons were more favourably oriented to repurposing while others to misuses. Informants noted that LLINs possessed certain qualities: versatile, strong, durable, multipurpose and aesthetically appealing. While most of these reasons generally linked to repurposing behaviours, the latter reason was a major motive for misuse reported behaviours. Poverty was another basic reason. Abject poverty and disappearing traditional working tools were considered as underlying reasons. While many of the reported behaviours were repurposing related, poverty was also a major reason for misuses (e.g. curtains, bed sheet and selling for cash support).

*Perception that LLINs can be used for other needs* was also an underlying driver, particularly for repurposing behaviours. As one elderly informant noted, “*We cannot just throw away or burn them down but we can use them for many needs*.” This driver is particularly related to perceptions of the life span of LLINs and in this case, it may be quite understandable. A saying from an old man supports this: “*when men get old, they go to the soil [as they die]; when LLINs get old they lose their original purpose*”. Improper uses and mismanagement often were major reasons for rapid wear and tear thus leading to repurposed uses. Informants noted that LLINs become old, torn and unfit for their intended purpose between mostly 6 months and 1 year and most users put nets for repurposed uses starting from 6 months’ span of service.

Furthermore, there are *reasons related to lack of awareness, knowledge and understanding:* Lack of knowledge, or awareness about the purpose and proper way of using the nets was reported by most informants. These were either due to some individuals’ own deficiency or to low level of training and education. *Individual differences in attitude, commitment and concern* were also reported as important reasons. Thus, certain individuals, even if they may possess good knowledge of the intended purposes, they often disregard this and continue using them for other purposes. Such people were more likely to engage in misuse behaviours such as selling for cash. *Wrong beliefs and perceptions* were other key reasons, often more likely the motives for misuses. Some persons believed malaria did not exist. Few others took malaria lightly arguing that it cannot as such harm or kill them and even if they get sick they can easily these days get treatments easily.

The residential pattern, living house conditions and arrangement make use of LLINs very difficult. The *inherent nature and technological features of LLINs* were also reported as conveniently designed in terms of their durability, size, colour and the like to attract misuses and repurposed uses. Some informants thought the way the nets are designed makes them difficult and cumbersome for some households. Thus, frustrations in being unable to use easily and simply often lead to misuse and repurposed uses.

**Fig. 7:**
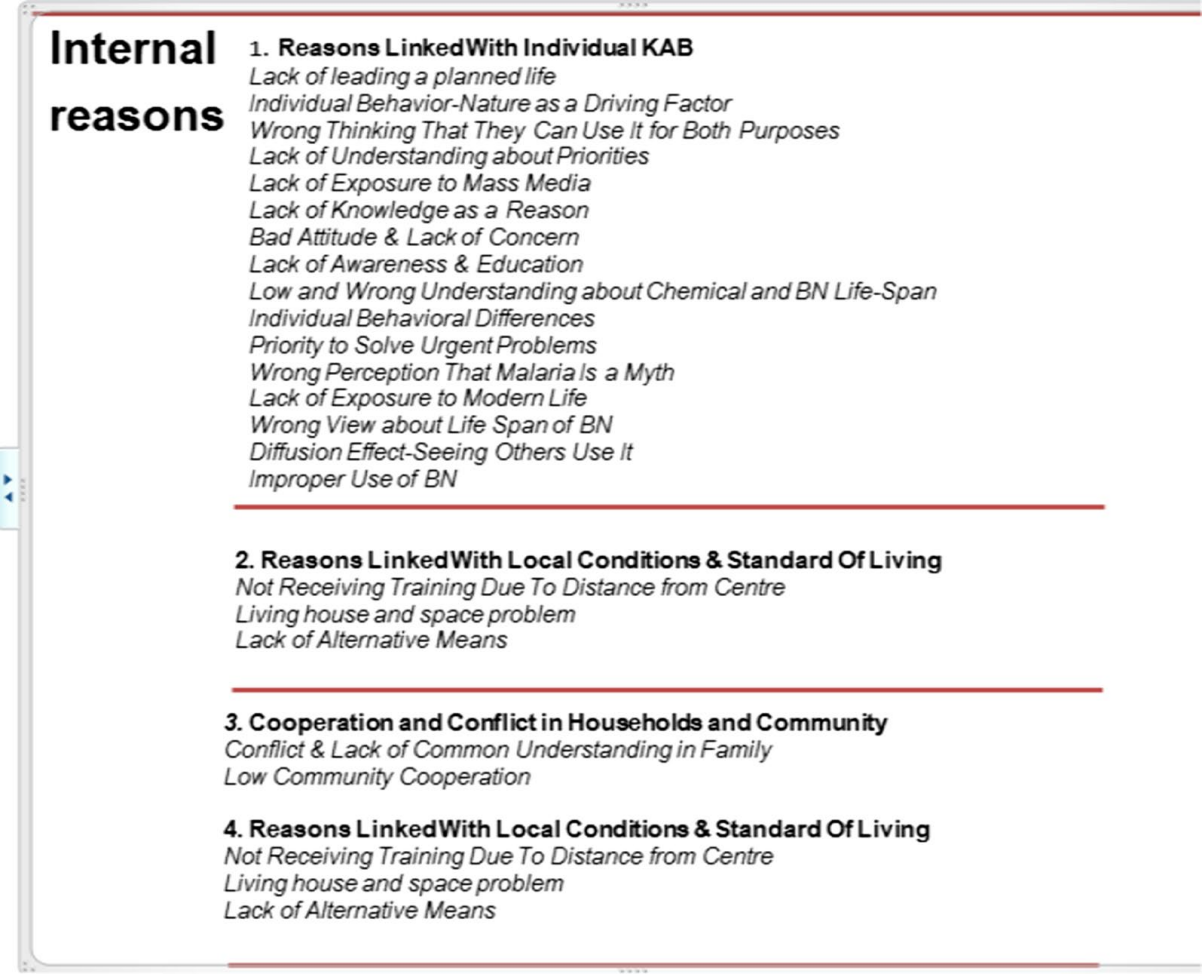
Summary of list of internal reasons for using LLINs for unintended purposes

**Fig. 8:**
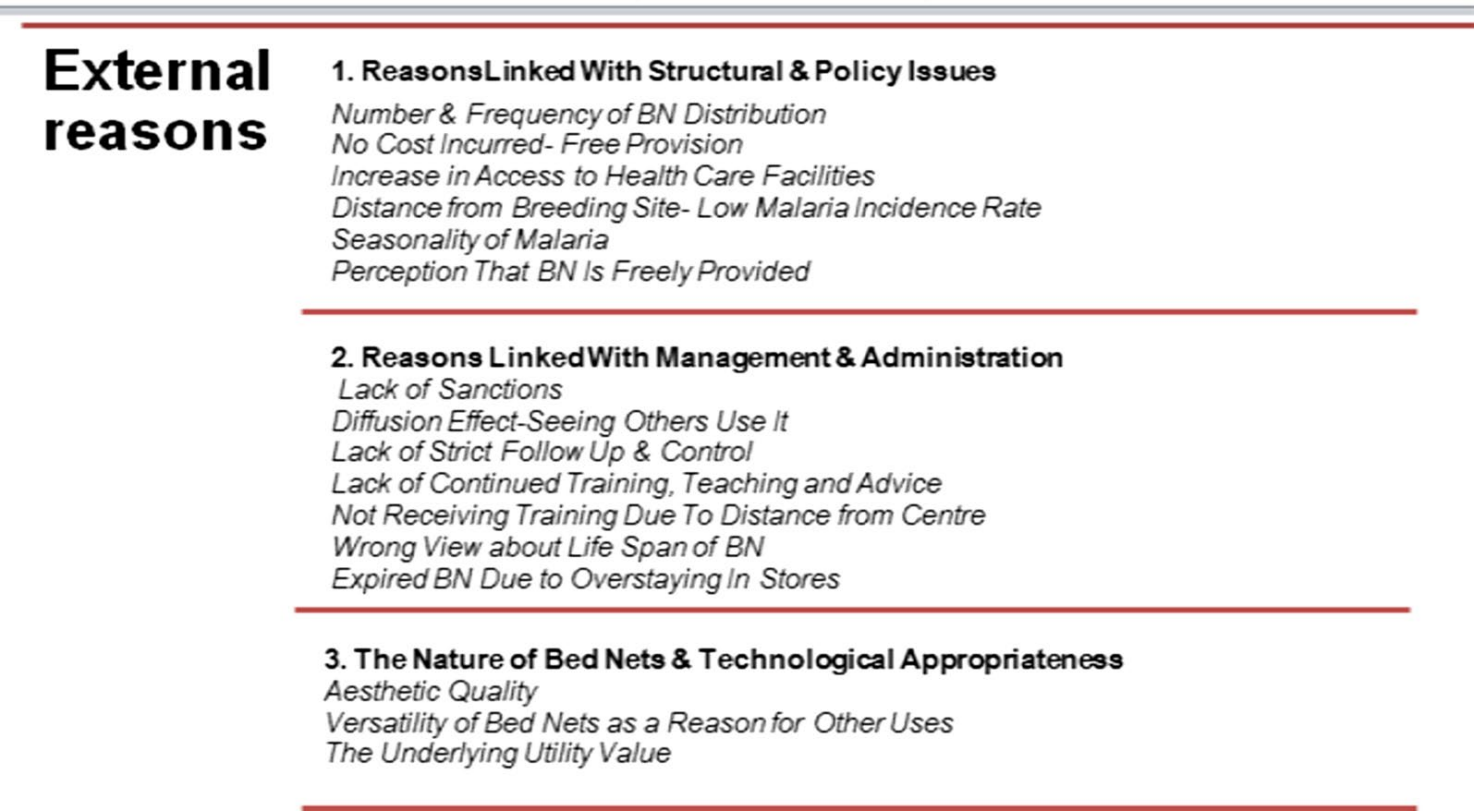
Summary of list of external reasons for using LLINs for unintended purposes

While the above reasons may be summed up as internal (Fig. 7), reasons related to *policy*, *management and administration issues* are what may be *termed external* reasons (Fig. 8). Health extension workers and users themselves reported that the policy of distribution, supply and cost sharing was also a factor to blame. Some households that received four nets simply misused spare nets (selling, giving away). As a health extension worker noted, providing nets free of charge being commendable, has also created an increasing sense of expectancy and dependence, contributing to low level of sense of ownership.

Issues of lack of effective coordination among concerned actors, lack of sanctions, community meetings, and strict follow ups, were most often raised as underlying external reasons that contributed to use of LLINs for unintended purposes.

Finally, reasons related to seasonality of malaria and distance from breeding sites were reported as other drivers. A health worker stated the fact that malaria is a seasonal health problem and locals often get lax about using the nets once the more malaria intense seasons elapses, arguing that its seasonality contributed to locals’ use of the nets for other purposes, mostly for repurposed uses. Distance from vector-breeding areas was also an important reason for proper use in some localities and misuses in other localities. While the researchers take this as an important factor in its own right in relating to peoples’ misuse or repurposed use of nets, they recognize it also as a proxy variable to malarial risk perception. The degree of strength of local peoples’ risk perception of malarial bouts may be thus considered as linked to how close or far households reside vis-à-vis the lake and irrigation canals.

### LLIN care and management

A very important factor behind the rapid decline in bed net ownership was the perception many individual users had concerning the *life span of insecticide* applied to the nets. While there was no explicit reference to the source of the information they had, most informants believed that after 6 months, LLINs lose their intended purpose because insecticides stop killing bugs. Another important set of drivers was *the lack of knowledge* in using the nets in proper ways, or failure to use them in proper ways even if they had knowledge about the types of cares required for the nets. Exposure of nets to sun, sharp things, mice, termites and child tampering, accounted for the rapid wearing and tearing of the nets.

Some of the drivers were external. The living condition and the type of residence are reported as major drivers of rapid wear and tear. Living in thatched grass house was mentioned as a key factor impeding proper care and use due to the lack of enough space, crowded living conditions and dirt. Dirt would often render the nets unfit for purpose within short period of time. Further, some users perceived that if the nets were washed several times this would lead to the rapid fading of the insecticide, making the nets unfit for the purpose.

### Division of labour in the household

Women informants summarily reported that only in very limited instances men in the household took active role and shared responsibility in the management of LLINs. Otherwise, invariably, it was the responsibility of women. Women further reported that if their men wanted to use LLINs for other purposes, they could and would often do it and that they had no power to say no. Only in about three cases that informants noted they could challenge if their husbands wanted to use LLINs for other purposes. Except some of the categories of other uses (such as curtain and head scarves) most of the other unintended uses of LLINs were “men stuff”. Men were reportedly more likely to misuse, for example, by selling for cash; it is men who repurpose the nets for ropes, fish nets, barn tops, storages, fence reinforcements.

## Discussion

In this section, the researchers discuss the findings under six salient themes: (1) use of LLINs for intended purposes; (2) factors affecting what is “right” about using LLINs; (3) knowledge gaps and misunderstanding about the LLIN purposes and lifecycle; (4) misuse of LLINs; (5) the repurposing of LLINs; and (6) gender dynamics in decision making regarding LLINs use.

### Use of LLINs for intended purposes

In the study community, use of LLINs for intended purposes in consistent ways was associated with a range of local perceptions and experiences regarding the benefits of LLINs. Firstly, the direct health benefit of preventing malaria was primarily important; but also, other social and economic issues were involved, including the benefit of low economic cost from reduced visits to health centres in relation to malarial problem. The qualitative findings highlight the locals’ apparent fascination with not just the direct, intended malarial protection benefit, but the collateral benefits including the repelling and killing of all sorts of insects such as bed bugs, fleas, flies, spiders, and other crawlers. Similar findings from a Uganda-based study also show the relevance of this, where Strachan et al. [14] report on the wider benefits of using nets, as experienced by targeted communities, beyond the prevention of malaria. They argued while the protection of malaria remains a powerful motivator, the non-malaria benefits of net use were also found very important in driving consistent use. Capitalizing on supporting this set of other non-malarial protection benefits of bed net use may, therefore, be an important factor in promoting the intended use of the nets.

The benefits derived from the use of LLINs especially in households with under-five and pregnant women was of paramount significance and some informants made an essential link to babies and pregnant women when describing the main intended purposes of LLINs. Families with children and pregnant women were more likely to use nets properly and the most immediate and widely recognized benefit of using nets thus centred on these demographic groups. However, it was not the case that the LLINs were distributed solely targeting such and other vulnerable sections of the population. The net distribution was based on family size and the national guideline. Studies conducted elsewhere in Ethiopia [7], Uganda [15, 16], and Western Kenya [8] in general attest that females and children having priority to sleep under LLIN with children between 0 and 4 years of age being more likely to use and that local people summarily prioritized pregnant women, infants, and young children when allocating LLINs.

### Factors affecting what is “right” about using LLINs, their care and utilization

LLINs’ utilization and care in Adami Tullu district were dependent on a range of factors, notably the local socio-cultural dynamics, residential patterns, distance of households from net distribution centres, and livelihood conditions. Comparable reasons were reported for communities elsewhere in Ethiopia [17, 18]; Kenya [19], Uganda [15], Rwanda [20], Nigeria [21], Mozambique [1] and Cameron [22]. All these studies demonstrate that following mass distribution campaigns, behavioural, demographic, environmental, sociocultural and livelihood factors were important in influencing ownership and proper use of LLINs.

Deep poverty issues prevailing in the communities were important above all in affecting people’s idea of what is right or wrong regarding net use, calling for addressing the underlying poverty. McLean et al. [23] forwarded similar observation based on their findings that underlying deep poverty was a factor in leading communities in Tanganyika to use LLINs in unintended/improper ways.

Apart from the broader socio-cultural and economic factors, individual behavioural and attitudinal dimensions were also important. While the general observation shows the community understanding about the nature and transmission mechanism of malaria is fairly strong, there were still certain lingering beliefs in the community about the mechanism of malarial transmission, with reports of some people holding the belief that malaria does not exist, or that malaria mosquito cannot harm them, which in turn influencing ideas about right use of nets. Perception of risk thus play important role. There are other risk perceptions that limit the importance of consistently and correctly using LLINs year round. For example, distance from vector-breeding areas was an important factor and perhaps it may be taken as a proxy for perceived risk of malaria infection. Households near such sites were observed and reported to use nets in proper ways more likely as they tended to hold stronger risk perception of malarial attack. The perception among some people that malaria mosquito cannot harm or cause death, or worse, that it does not exist, was another risk perception which further bar people from using nets all year round.

Such beliefs and ignorance about mechanisms of malaria transmission and proper use of the nets were reported in Ethiopia and elsewhere and in Africa, showing the links between an individual’s knowledge and beliefs related to malaria and LLINs (see for example [22, 24–28]). Githinji et al. [29] for Kenya, and Berhanu et al. [7] for Ethiopia similarly reported that behavioural dynamics such as low risk perception, saving nets for future use, awareness and negligence accounted for failures in proper use of LLINs.

A related factor is the perceived and experienced versatile usability and technological design of nets creating operational inconvenience influencing peoples’ ideas about proper use and care of the nets. This emerged as an important issue in the Adami Tullu study, a fact echoed also in other studies. For example, Fuge et al. [27] in Ethiopia; Ernst et al. [8] in Malawi; Kateera et al. [20] and Ingabire et al. [30] in Rwanda, all reported lack of awareness on how to install LLINs and challenges in using LLINs, including shape, inconvenience, heat, and discomfort; difficulties in proper net hanging, appearance or ability to keep it clean, and the perception that it should be used only with a bed. In view of this, McLean et al. [23] argued about introducing new technologies and suggested introducing new technologies such as changing from the moveable LLINs’ prevention to immovable wall painting which are easy to mobilize for other uses, to something stationary, such as wall treatments or newly developed individual spatial repellents.

A further important external factor influencing peoples use and care of LLINs was the issue of free supply of nets. There was a widespread sense of dependency among the locals on the external support and guidance, suggesting a need for making the users share the cost of LLINs and enhancing their awareness about their own duties in maintaining and caring for the nets. In other comparable studies, some findings suggest the need for reducing community dependence on free distribution, through promoting the idea of on-going net care and replacement as a household responsibility [14]. Strachan et al. [14] further suggest the promotion of viable, affordable options for the replacement of nets where the supply is available. This can include introduction of cost-sharing policy, among others, whereby locals can be convinced to contribute some token proportion of the cost of nets.

There were diverse positions, though, regarding the free distribution of LLINs. Some studies suggest more focus on making net available freely in greater scale [16]. Mass distribution of free LLINs is generally positively regarded and found contributing positively towards ownership and use [31]. Unlike many African countries, the distribution of nets for free has only been introduced recently in Ethiopia through WHO, UNICEF, NGOs and Ministry of Health (Jima et al. 2009, cited in Fetene et al. [25]). Studies conducted in Cameroon [22] and Uganda [16] highlighted the need for more focus on scaling up free provision of nets for poor households, to address issues of equity and meet the needs of households that could not use nets for economic reasons.

In general, as Russell et al. [21] argue, malaria protection will only be achieved if LLINs are used both correctly and consistently, as accessibility and ownership do not automatically equate with proper use, an outcome strongly shaped by complex local socio-cultural, economic, environmental and demographic realities.

### Knowledge gaps and misunderstanding about purposes and lifecycles of LLINs

Coupled with the underlying socio-economic, cultural and behavioural variables influencing ideas about right use, lack of knowledge and low proper training provided upon provision of nets were important factors contributing towards misunderstandings about net life cycle in the study areas. Local perceptions of effectiveness of nets often importantly related to the ability of the insecticide to kill insects when the nets are regularly used and washed for a number of times. The rampant shared understanding in Adami Tullu community was that LLINs stopped being effective after 6 months to 1 year because “they no longer kill insects”. Such perceptions were reported elsewhere in Ethiopia [25], Ghana [32] and Kenya [8], where informants questioned the ability of LLINs to withstand regular use and once the nets no longer were able to kill other insects they felt the nets were not effective for killing mosquitoes.

In general, the effect of the insecticide treatment on the other insects had an important effect in the users’ willingness to continue using the nets for the intended purposes or else putting them for other purposes [23, 24]. Further, people did not use their nets for the recommended service years. The World Health Organization recommends 3-year LLIN serviceable life span [26]. The view that LLINs were expected to serve for at least 3 years was not understood among users in Adami Tulu; neither did most of the interviewed users believe the nets should serve such length. In view of this, it was not surprising that some local informants suggested that the expected service year of the nets should be not more than 2 years, a fact confirmed by findings from other studies (see for example, [26]).

It is worth-noting here that while the origins for the wrong perceptions about the proper use, care and maintenance of LLINs and their short life cycles may be attributed to individual behaviours and attitudes, there was also a firm reference to the gaps in knowledge dissemination on the part of the project staff. Such gaps are generally reported as rampant across communities in Africa. Studies conducted in Bui, Ethiopia [25], Uganda [16], and Kenya [8] underscored that local users had superficial knowledge on malaria and its transmission, including the use and maintenance of LLINs.

Education, communication and follow up were reported as glaringly low in our Adami Tullu study. Locals reported, to be fair, education and trainings were provided for users and home visits by health extension workers were made. However, there was a gap in the way messages and communications about life-cycle of LLINs and their use and care were communicated to the users. Studies on behaviour and social change strategies show that messages and their communications can have positive and negative impacts depending on their appropriateness and effectiveness. As Owusu [33] argued, efforts to relate LLIN messages to the public are very useful in increasing use of LLINs. The lack of consistent, systematic and tailored information, communication and education provision was a problem in much malaria prevention and LLINs utilization matters in Ethiopia [27], Côte d’Ivoire [5], Kenya [8] and Mali [34].

In Adami Tullu as well as in these countries, local users complained about lack of proper training, that nets were in some cases simply supplied without any training on their purposes and how to use them. Sensitization of households to use LLINs through regular home visits, and promoting informal and formal social support networks produces positive outcomes in terms of proper use [5]. This calls for the importance of community outreach, interpersonal communication and social support [21].

### The repurposing of LLINs

Studies reporting on why people employ LLNIs for repurposed uses and types of such uses are emerging [8]. Reported and observed behaviours relating to repurposing were growing phenomena in Adami Tulu. Similar studies elsewhere in Ethiopia and Africa report that local users engage in using nets for repurposed uses. Studies by Gobena et al. [10] in Ethiopia, Ernst et al. [8] in Kenya, Ingabire et al. [30] in Rwanda, and Koenker et al. [35] in four countries of Nigeria, Ghana, Senegal and Uganda, all have reported that people used bed nets for other purposes, notably repurposed uses.

The Adami Tullu study demonstrated that the most common types of repurposed uses of nets were linked to the prevailing socio-cultural and livelihood conditions. Such uses particularly peaked the most during corns harvest season in Adami Tulu. Fishing was an important activity in some localities nearer to the lake and nets were used routinely for fishing purposes. The other common uses such as roping, wall plastering and fencing were all linked to local socio-economic conditions. In a comparable way, other studies have documented the most common repurposed uses of nets were fishing nets, ropes and fencing. LLINs were used in constructing chicken pens in Ethiopia [10]; for fishing in the Tamatave region of Madagascar, and in Lake Tanganyika [23]; for fences around vegetable gardens in Rwanda [15], a range of other repurposed uses in costal Kenya [36]; and for drying fish near Lake Victoria [37].

The question of when do people employ nets for repurposed uses is important. While in very limited instances informants reported the use of supplied nets in their new and serviceable life cycle stages (see below), it was generally established that people employed nets for repurposed uses after they determined that the nets had ceased to offer their intended purposes due to old age, or perceived loss of power to kill mosquitoes. Koenker et al. [35] explored what happens to lost nets and at what stage of life cycle do people apply nets for re-purposed uses. They noted that of the repurposed nets, the majority were already considered too torn, indicating they had already served out their useful life for malaria prevention.

Perception about the multi-purpose uses of LLINs was equally an influential factor in the repurposed uses of the nets. Local understanding of LLINs as versatile, broad-spectrum usability and function were important reasons of why people use nets for other purposes. Comparably, a study from Kenya reported local informants’ speaking about a bed net is a multi-purpose item [8].

Some informants in Adami Tulu study noted that free distribution of the nets was a key motivation for using the nets for other purposes, particularly for repurposed uses (though this also motivated misuse). A study by McLean et al. [23] on the use of ITNs for fishing purposes in Tanzania reported that all of the surveyed informants used LLINs for fishing proposes at some point and that over 90% had received the nets freely, implying that the free supply of the nets might have served a motivator for engaging in such purposes. It is a general assumption that where something is freely obtained people tend to not feel pain when they misuse it. If they are made to share the cost, they would behave differently. Studies suggest that introduction of cost-sharing scheme can be run side with free distribution campaigns, whereby those households relatively able may be made to participate in the cost sharing scheme. This can encourage more responsible use and care. Eze et al. [31] report some success story from Tanzania where a voucher scheme was used as a form of subsidy by the government.

### Misuse of LLINs

In the Admai Tulu study, the fact of misusing nets was obviously established through locals themselves acknowledging such uses, reporting their having used it themselves, seen other using it and also such uses being demonstrably observed by health workers. Informants were also pointedly remorseful for their community being implicated in using nets in their new states for other purposes.

There were certain motivating factors for why individuals chose to misuse an LLIN. A major reported motivation was related to distribution and supply policy which reportedly encouraged such misuses. While households with pregnant women and babies were more likely to use nets properly; conversely, households without such were usually apt to misuse them. Malarial risk perception which tended to be stronger among households residing near breeding sites motivated proper use while low risk perception among households living farther away from such sites encouraged misuse. Use of nets in new states was similarly found among other communities in Ethiopia and elsewhere in Africa. A study of the use of mosquito nets in a malaria endemic region in south western Kenya by Githinji, Herbst and Kistemann showed that nets in their new state were diverted to other uses such as table clothes, wall hanging and curtains [29]. Similarly, Eisele et al. reported on misuse of nets [38].

Problems with net distribution arrangement often encouraged misuse. The provision of many LLINs based on the size of households was described by some locals as a factor that played a role in the way some households misused nets. Some novel net distribution strategies might, as, for example, one reported by Plucinski et al. [39] in their Mozambique-based study, where sleeping arrangement within household was combined with size of household when distributing nets, may be employed to address this problem.

### Gender dynamics in decision-making regarding LLIN use

The findings in this paper raise some interesting gender dynamics on the role of men and women in decision making roles for use of LLINs. An important socio-cultural factor involves *the dynamics of division of labour and decision making* related to the use, misuse or repurposed use of LLINs. Women informants generally held the view that male household heads had a swaying level of decision making with respect to LLINs. Whether hang, care for, sell or use the nets for other purposes are influenced by existing social and cultural dynamics. Comparably, in Tanzania, household decisions to keep, hang and use net or using it for other purposes were dependent on cultural norms in which males hold decision making power while net administration becomes a woman’s thing [40]. One important finding of Adami Tullu study was in households where there was fair cooperation among household members, particularly between husbands and wives, proper use of LLINs was more likely. Husbands need to be made active agents in the enhancement of proper behaviours with respect to net use. Elsewhere in Africa, a study by Strachan et al. [14] show the need for such behavioural and social strategies; i.e., the importance of including male household heads and caregivers as key agents in sustaining net use behaviour. As Ricotta et al. [40] argue, besides the problem of acquiring LLINs, behavioural change communication strategies addressing such dimensions will, therefore, help generate desired positive behaviour and decision outcomes for gender balance in decision making powers and shared responsibilities to engender proper use of nets and avoid their misuse.

## Conclusion

Behavioural, socio-cultural, economic and ecological conditions coupled with deficiencies in perceived bed net design and distribution policies and weak education, communication and social support structures were important in understanding and accounting for why a low level of intended use and a rampant misuse and repurposed use in Adami Tullu community of Ethiopia. A major nexus for addressing so as to improve intended use of LLINs lies, first and foremost, are the economic poverty and socio-cultural factors that underlie much of the misuse and repurposed use of the nets.

In sum, it can be concluded that (1) certain households and users were more likely to use nets for intended needs in proper ways; (2) a range of factors, notably socio-cultural and poverty, highly influence users’ ideas about the right ways and decisions to use and care for the nets; (3) knowledge gaps and wrong perception exist regarding the purposes and life cycles of the nets; (4) LLINs are employed for repurposed uses once they are considered non-viable, old, or lose their physical integrity; (5) existence of misuse was acknowledged and understood as wrong; and (6) values about gender roles further shape uses, misuses and repurposed use of the nets.

There are significant opportunities for SBCC interventions to improve the understanding of and behaviour towards LLIN use. There were tangible concerns as quite many users believed the nets did not serve beyond 1 year; similarly many struggled as how best to care for and manage the nets; some informants also thought they were not at risk of malarial attack. In view of this, then it is important that proper training and education be provided for those that are particularly prone to hold wrong perceptions about malaria; provide similar trainings supported with demonstration on the proper life span of the nets, and appropriate ways of caring for the nets.

### Limitations

While the analysis of 86 (individual and focus group) interviews offers insight into rural community-based perceptions of LLINs, the researchers recognize the limitations of the study and the conclusions are framed to focus on appropriate local solutions to the issues found in the qualitative study. The researchers understand the results may not be generalized into recommendations about the design and structure of LLINs themselves.
